# Immediate prosthesis over implants retained using abutments with flexible screws: A preliminary study

**DOI:** 10.4317/jced.53806

**Published:** 2017-12-01

**Authors:** David Peñarrocha-Oltra, Blanca Serra-Pastor, José-Carlos Balaguer-Martí, Miguel Peñarrocha-Diago, Rubén Agustín-Panadero

**Affiliations:** 1 Assistant Professor. Oral Surgery Section, Department of Stomatology, Valencia University Medical and Dental School. Valencia, Spain; 2 Master in Prosthodontics. Department of Buccofacial Prostheses, Complutense University Dental School. Madrid, Spain; 3 Master in Oral Surgery and Implantology. Department of Stomatology. Valencia University, Medical and Dental School, Valencia, Spain; 4 Full Professor. Director of the Master of Oral Surgery and Implantology. Oral Surgery Section, Department of Stomatology, Valencia University Medical and Dental School. Valencia, Spain; 5 Associate Professor. Department of Stomatology, Faculty of Medicine and Dentistry, Valencia University, Spain

## Abstract

**Background:**

Immediate loading protocols for the rehabilitation of edentulous or partially edentulous patients have become very popular, due to the conveniences they afford in comparison with conventional loading techniques.

**Material and Methods:**

A preliminary study was carried out with 8 patients subjected to dental implant treatment with an immediate loading protocol involving a novel system of abutments with flexible screws. Implant survival was analyzed, together with marginal bone loss and patient and dentist satisfaction.

**Results:**

A total of 35 implants were subjected to immediate loading using the abutments with flexible screws. The mean patient and dentist satisfaction score was 9.1 and 8.5, respectively. After 12 months the dental implant survival rate was 95.8%, with a mean marginal bone loss of 0.51 ± 0.12 mm.

**Conclusions:**

The novel system of abutments with flexible screws offers a good alternative to conventional immediate loading, since it allows rapid and simple manufacture of a reliable passive fit, fixed interim prosthesis after surgery.

** Key words:**Dental implants, Flexafit®, Immediate loading, Immediate prosthesis.

## Introduction

Immediate loading protocols for the rehabilitation of edentulous or partially edentulous patients have become very popular, due to the conveniences they afford in comparison with conventional loading techniques.

The waiting periods from implant placement to prosthetic loading have been reduced as a result of the introduction of new implant surfaces and designs, and immediate implant loading is currently possible in selected cases. The definition of immediate loading has evolved in the period between publication of the Barcelona consensus document in 2002 (1), where it was taken to represent loading in under 24 hours, and the Cochrane review published by Espósito *et al.* in 2007 (2), where the immediate loading limit was defined as one week after surgery.

Immediate loading offers a series of advantages with respect to delayed loading, including the provision of immediate aesthetics and function, elimination of the need for a removable interim prosthesis, and the avoidance of second surgery. When combined with immediate implant placement, it moreover avoids an edentulous period for the patient (3,4). The immediate prosthesis may be screw-retained or cemented, though cemented prostheses can give rise to biological complications affecting the peri-implant tissues if the excess cement is not correctly removed (5). This problem does not occur in the case of screw-retained prostheses, though the latter are associated to an increased incidence of prosthetic and biomechanical complications such as difficulties in obtaining correct passive fit, or possible fracture of the prosthesis or of prosthetic accessories such as screws (6).

Immediate loading protocols also have a number of inconveniences, such as the difficulty and time involved in producing an implant-supported interim prosthesis, or deformation of many of the commonly used plastic abutment materials as a consequence of time and loading (7).

An immediate loading protocol has recently been described involving an innovating system of prosthetic accessories that facilitate the procedure, minimize its inconveniences (e.g., technical difficulty and clinical working time), and make aspects such as the obtaining of impressions and laboratory steps easier (8,9).

We present a preliminary study of 8 patients subjected to dental implant treatment with an immediate loading protocol involving a novel system of flexible accessories, allowing fitting in the dental clinic of a screw-retained fixed prosthesis immediately after implant placement. Evaluation was made after one year of follow-up, since there is practically no clinical evidence on the use of this system.

## Material and Methods

In this preliminary study we treated a series of patients using an immediate loading protocol with Avantblast surface Phibo TSH® external connection implants (TSH® implants, Phibo Dental Solutions, Barcelona) and the Flexafit® prosthetic system (TFACS DE Flexafit®, Dentisel, Bellavista, Barcelona). The study was carried out in the Oral Surgery Section of the Department of Stomatology (Valencia University Medical and Dental School, Valencia, Spain) between January 2014 and December 2015.

2.1 Study design

The following inclusion criteria were used in selecting cases amenable to treatment with immediate loading: non-smoking patients presenting ASA score I and aged over 20 years, implants with primary stability > 35 N, and subjects with the placement of at least three implants.

The patients were treated between January 2014 and December 2015, and were enrolled in a prospective cohort. The study was approved by the Ethics Committee of the University of Valencia.

2.2 Description of the procedure

**Figure 1:**
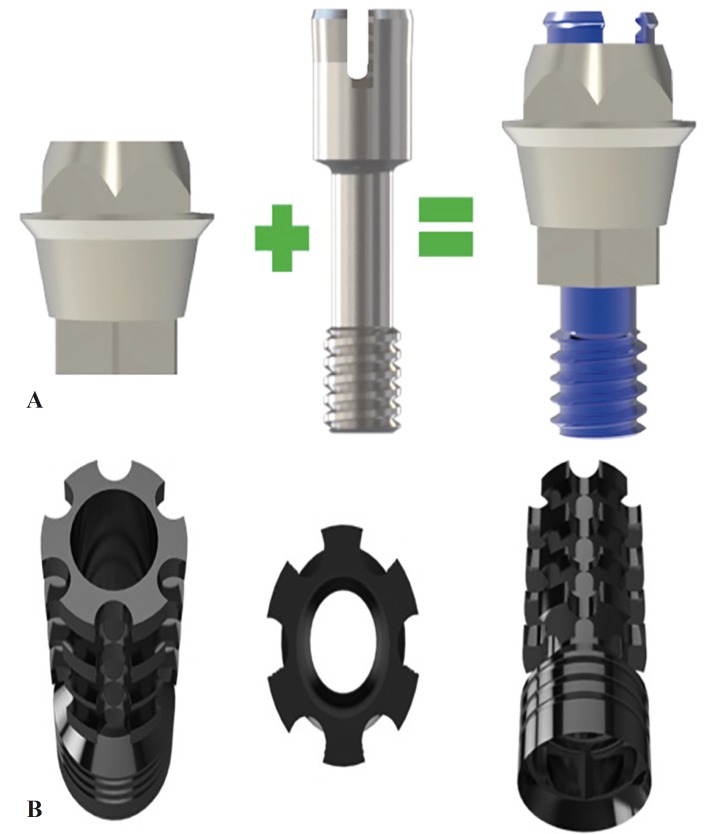
A) From left to right: primary abutment, flexible screw with grooves, abutment and screw assembly. B) Secondary abutment, frontal and occlusal view.

Immediate loading was carried out based on the Flexafit® system protocol described by Balaguer *et al.* (9). The Flexafit® system consists of a primary abutment made of grade V titanium compatible with all implant connections currently found on the market. The upper part of the primary abutment together with the head of the screw conform the universal connection that characterizes the system, and on which the secondary abutment, also made of grade V titanium, is positioned. The special characteristics of the screw of the primary abutment afford a degree of flexibility (Fig. 1a). The head of the screw stands out from the primary abutment and has three grooves that allow connection with the secondary abutment by means of a snap-on (connection with pressure) metal-to-metal mechanism. Lastly, the head of the screw also has an internally threaded perforation allowing screw-retention of the secondary abutment (Fig. 1b) after snap-on adjustment.

The system offers different primary abutment heights to facilitate placement of the secondary abutment for immediate prostheses in those cases where the implants are positioned at subcrestal level or in patients with excess soft tissue. It also should be mentioned that the different primary and secondary abutment widths make it possible to correct disparallelisms for immediate loading of between 30-60 degrees.

In all cases, the surgical part of the treatment was carried out by three dentists specialized in implantology, while the procedure of immediate loading and subsequent manufacture of the definitive prosthesis was performed by two prosthodontists.

**Figure 2:**
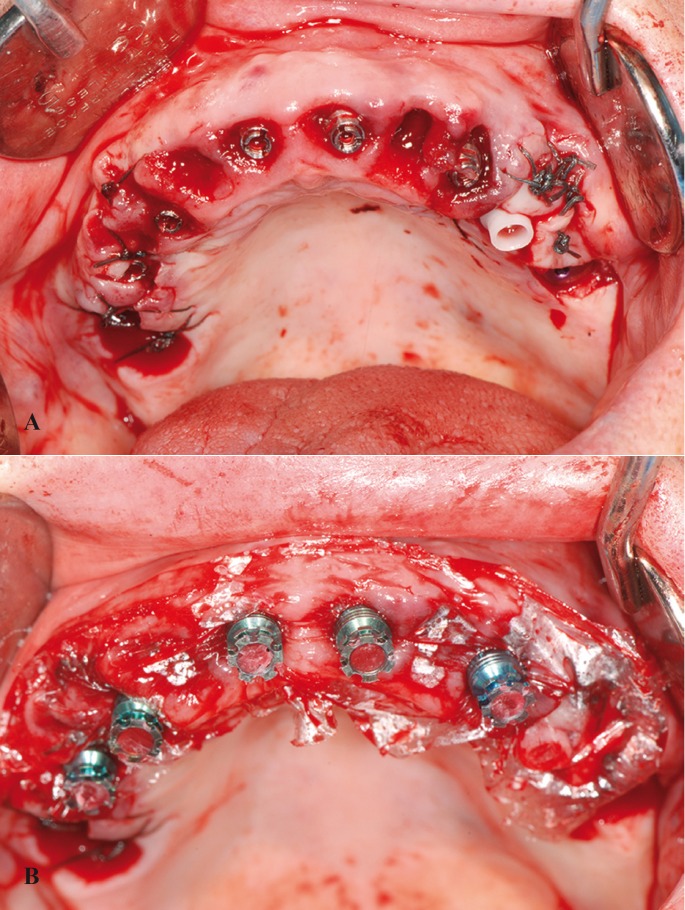
A) Primary abutments positioned after tooth extraction and posterior implant placement. B) Placement of the thin protective plastic film and interim secondary abutments on the primary abutments. Blocking of the secondary abutment holes before rebase of the prosthesis.

**Figure 3:**
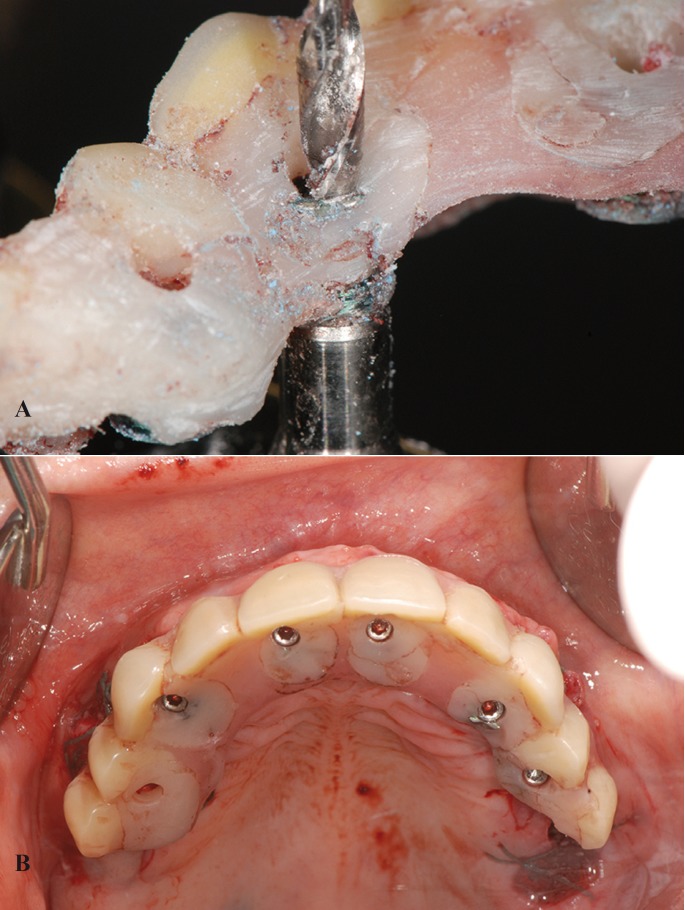
A) Perforation of the interim prosthesis using the Flexafit® drill. B) Screw-retained interim prosthesis in mouth, occlusal view.

The following steps were followed to implement the Flexafit® system protocol for immediate loading:

1. The primary abutment was connected to the implant with a torque of 30 Ncm (Fig. 2a).

2. A thin protective plastic film was positioned over the primary abutment in order to isolate the gums and abutment from the acrylic resin of the rebase of the prosthesis (Fig. 2b).

3. The interim grade V titanium secondary abutment was fitted onto the primary abutment by means of the snap-on mechanism, without screw retention. The hole of the secondary abutment was filled with wax to prevent the penetration of rebase resin.

4. The interim prosthesis was rebased with acrylic resin (Sintodent®, Sintodent S.R.L.; Rome, Italy).

5. The holes for access of the prosthetic fixation screws were prepared through the secondary abutments (Fig. 3a). To this effect, the base of the secondary abutment was fitted onto a special accessory with the shape of the head of the primary abutment and mounted onto the Flexafit® precision drill, which is equipped with a 2 mm diameter tungsten carbide bur for perforating perpendicular to the base of the connection.

6. The prosthesis was drilled, polished and prepared to ensure correct anatomical characteristics for adequate patient-performed hygiene.

7. The interim prosthesis was fitted onto the primary abutment, and stability was ensured by tightening the fixation screws at a torque of 20 Ncm (Fig. 3b).

8. An X-ray was obtained to check passive fit.

After placing the interim prosthesis, dentist satisfaction with the Flexafit® system was evaluated based on a 10-cm visual analog scale (VAS).

One week after the operation the patients were evaluated, and their general satisfaction was likewise scored using a VAS.

Two months later, prosthetic rehabilitation was performed using CAD/CAM techniques for manufacturing a fixed full-arch, implant-supported rehabilitation. From the STL files obtained by the extraoral scan, the laboratory technician used a CAD tool (3Shape CAD Design Software, Copenhagen, Denmark) to design the direct to implant framework made of cobalt-chrome (Ad-hoc®, Phibo Dental Solutions, Barcelona).

2.4 Follow-up and study variables

The patients were followed-up on one week and one, 6 and 12 months after immediate loading. We recorded age and gender, as well as the level of oral hygiene and the location and length of the positioned implants.

Mean satisfaction of both the patients and dentists was scored using a VAS 10. In the case of the two dentists participating in the procedure, satisfaction was explored by means of three questions: ease of handling of the system, the time required to complete the procedure, and aesthetic outcome. Patient satisfaction in turn was evaluated by 6 questions that rated general satisfaction with the implant-retained prosthesis, comfort and stability of the prosthesis, speech, ease of hygiene, aesthetics and function ( Table 1). The VAS of both the dentists and patients scored the different items from 0 (totally dissatisfied) to 10 (totally satisfied).

**Table 1:**
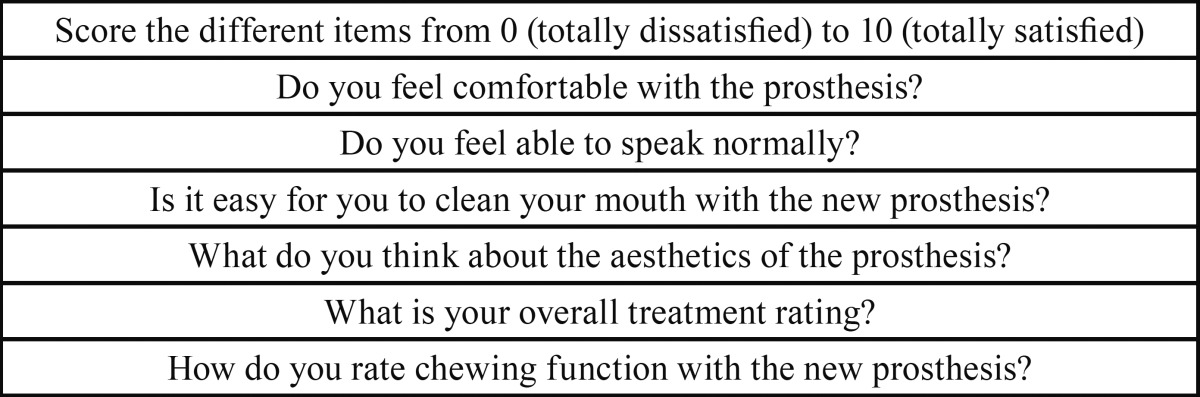
Patient satisfaction questionnaire.

Implant survival and peri-implant marginal bone loss were assessed one year after loading, using parallelized periapical X-rays obtained at the time of loading and one year after loading. Peri-implant marginal bone loss was assessed using the DBSWin® application (Dürr Dental, Bietigheim-Bissingen, Germany), establishing two arbitrary points at the crown-implant junction to determine a straight line. Two straight lines were then traced perpendicular to this first line both mesial and distal to the implant. The difference between the measurements obtained before and after loading defined bone loss – the highest value among those calculated mesial or distal being considered in all cases (11).

## Results

Eight patients with a mean age of 60.8 years (85.7% women) were treated with the Flexafit® system during the study period. A total of 35 post-extraction implants were placed (four upper maxillary and four mandibular restorations). Full arch prosthetic restorations were carried out in three cases, while fixed partial prostheses were placed in 5 cases. The three full arch restorations were all in the upper maxilla on 6 implants. Four fixed partial prostheses were placed in the mandible: all comprised four crowns on three implants in the anterior sector, with the exception of one 6-crown restoration on four implants. A single fixed partial prosthesis comprising 6 crowns on three implants was placed in the upper maxilla. Both the full arch and the fixed partial restorations were subjected to immediate loading with occlusal contacts starting on the day of placement.

The lengths of the 35 implants were: 10 mm in 5 cases, 11.5 mm in four cases, 13 mm in 14 cases, and 14.5 mm in 12 cases. The implant survival rate was 95.8%, since one implant in a fixed partial prosthesis in the mandible failed one month after loading. Lastly, the mean marginal bone loss was found to be 0.51±0.12 mm ( Table 2).

**Table 2:**
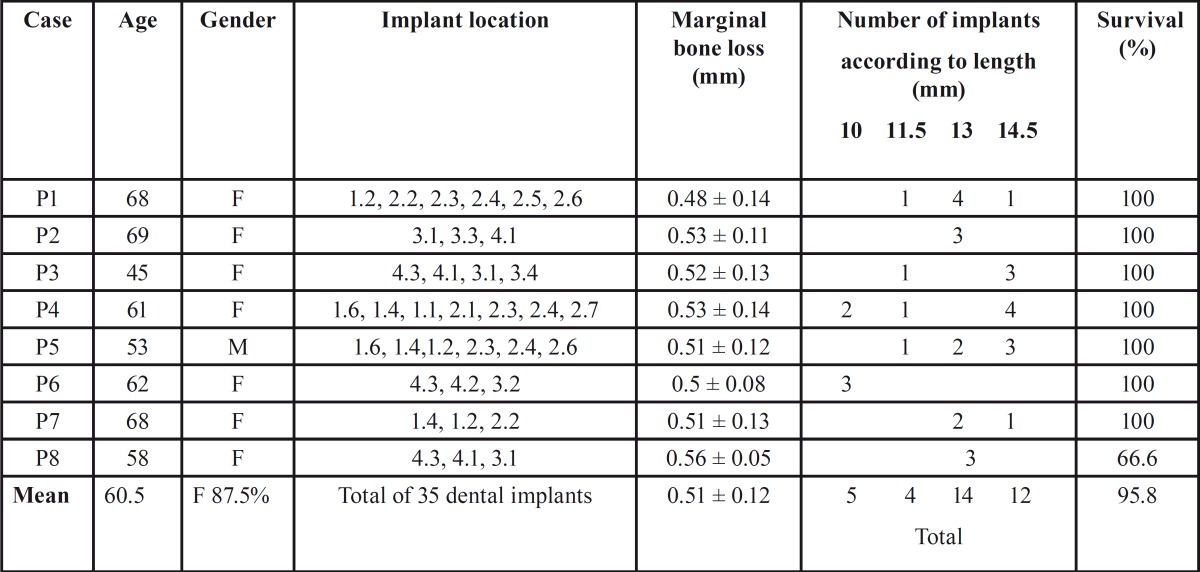
Description of the cases.

The mean dentist satisfaction score (VAS) with the Flexafit system was 8.5 ( Table 3). Overall patient satisfaction with the immediate loading treatment was also high, with a mean score of 9.1. However the score given by the patients to ease of hygiene was much lower, with a mean value of 6.6. Patients who scored the worst were those with complete arch prostheses (scores between 5-6), in contrast to patients with fixed partial dentures, who yielded scores between 7-8 ( Table 4).

**Table 3:**
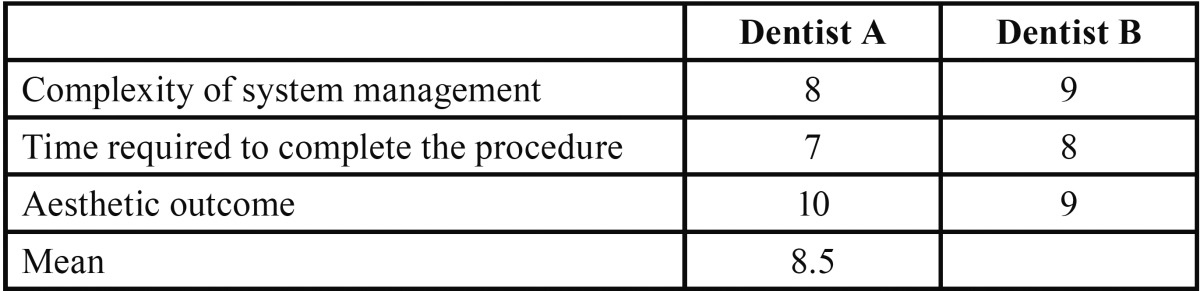
Dentist satisfaction with the Flexafit® system.

**Table 4:**
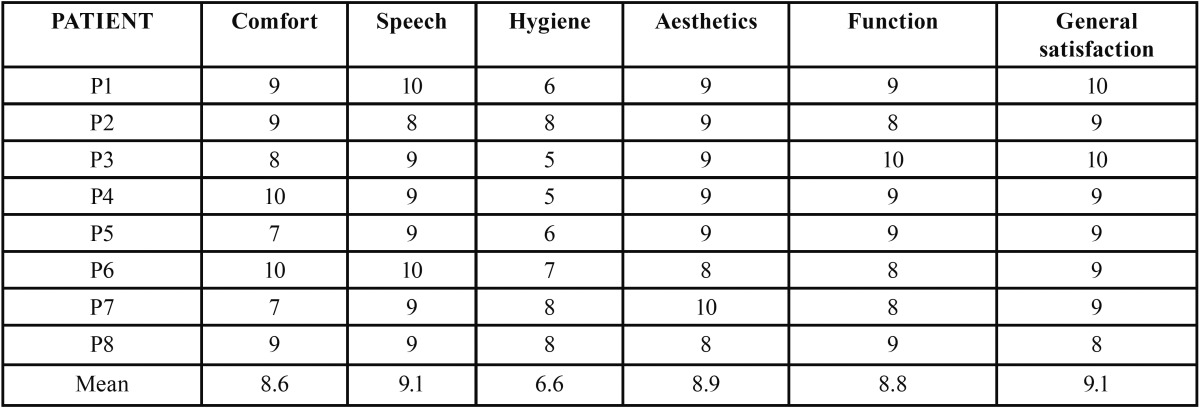
Patient satisfaction with the treatment received.

## Discussion

The manufacture of interim prostheses for immediate loading in implantology is generally a long and complex process. The present study has analyzed a new system that allows simple and rapid manufacture of interim prostheses. There are two general options for implant-based restorations: cemented and screw-retained prostheses. The studies that have compared both options have reported no statistically significant differences in bone loss after 10 years between the two techniques (12,13). However, failure to eliminate the excess cement in cemented prostheses can result in peri-implant tissue irritation and marginal bone loss (14,15). The use of a screw-retained prosthesis therefore eliminates one possible risk factor for peri-implant bone loss.

The implant survival rate in our series was 95.8%, which is consistent with the results published by Balshi *et al.* (16) and Cannizaro (17), who recorded a 99% survival rate in their conventional immediate loading studies. The three failed implants in our preliminary study corresponded to one same patient with a fixed partial prosthesis – failure being attributed to poor oral hygiene and the presence of parafunctional habits. The marginal bone loss was 0.51 mm, which is consistent with the data found in the literature (18,19).

Rehabilitation with dental implants subjected to immediate loading offers a series of advantages with respect to delayed loading or removable prostheses, including the provision of immediate aesthetics and function, elimination of the need for a removable interim prosthesis, with the psychological advantage that the patient is not left edentulous at any time, and the avoidance of second surgery (4). The Flexafit® system allows easy and rapid manufacture of the interim prosthesis, shortening the working time and guaranteeing correct passive fit thanks to the snap-on (pressure) metal-to-metal mechanism, which moreover allows us to check the emergence profile without having to continuously screw and unscrew the interim prosthesis. This is an important advantage, since it allows the procedure to be carried out immediately in the clinic after implant placement, without having to resort to the laboratory technician. On the other hand, the advantages of a screw-retained prosthesis are maintained, since a drill is used after completing the interim restoration to perforate the holes and screw it onto the implant.

General satisfaction among the patients and dentists participating in the study was assessed using a visual analog scale, as in other publications (10). The scores for general satisfaction were high in both cases: 9.1 among the patients and 8.5 in the case of the dentists. In the case of the dentists it should be noted that the lowest score corresponded to the time needed to carry out the procedure. This can be explained by the fact that the operators were not yet familiarized with the Flexafit® system. Furthermore, they were not used to performing immediate loading entirely in the clinic, without intervention by the laboratory. With regard to patient satisfaction, ease of hygiene of the prosthesis scored poorly, particularly in those patients with a full-arch prosthesis. This can be explained by the fact that these are screwed fixed prostheses in which increased prosthesis size is associated to a greater probability of food particle retention and to more difficult hygiene.

One of the limitations of the technique is the need for a precision drill specific of the Flexafit® system for preparing the holes used for screw-retention of the prosthesis in a simple way and without the risk of damaging the secondary abutments. Another limitation is the need for a degree of skill on the part of the dentist in order to obtain an interim prosthesis with optimum aesthetic and polishing conditions, since the procedure is designed to be carried out in the clinic.

Lastly, mention must be made of the fact that our preliminary series involved a limited sample size; studies with a greater number of cases are therefore needed in order to obtain more solid results.

## Conclusions

Despite the limitations of our study, the results obtained suggest that the flexible abutment system is a good alternative to conventional immediate loading, since it allows easier and faster manufacture of the interim prosthesis thanks to connection and removal via the snap-on (pressure) metal-to-metal mechanism, and at the same time ensures a good final passive fit.
